# Development of the Fearless, Tearless Transition model of care for adolescents with an intellectual disability and/or autism spectrum disorder with mental health comorbidities

**DOI:** 10.1111/dmcn.14766

**Published:** 2020-12-17

**Authors:** Evelyn Culnane, Hayley Loftus, Daryl Efron, Katrina Williams, Nikki Di Iorio, Rebecca Shepherd, Catherine Marraffa, Lionel Lubitz, Giuliana Antolovich, Chidambaram Prakash

**Affiliations:** ^1^ Transition Support Service Department of Adolescent Medicine The Royal Children’s Hospital Melbourne VIC Australia; ^2^ Department of Paediatrics University of Melbourne Melbourne VIC Australia; ^3^ Health Services Murdoch Children’s Research Institute Melbourne VIC Australia; ^4^ Department of General Medicine The Royal Children’s Hospital Melbourne VIC Australia; ^5^ Centre for Community Child Health The Royal Children’s Hospital Melbourne VIC Australia; ^6^ Department of Paediatrics Monash University Melbourne VIC Australia; ^7^ Department of Mental Health The Royal Children’s Hospital Melbourne VIC Australia; ^8^ Department of Neurodevelopment and Disability The Royal Children’s Hospital Melbourne VIC Australia

## Abstract

**Aim:**

First, to understand the barriers to achieving effective transition and the supports required from the perspective of parents and carers, adolescents with intellectual disability and/or autism spectrum disorder and co‐existing mental health disorders (often termed ‘dual disability’), and those who provide services to this group. Second, to develop an informed model of shared care to improve the transition of adolescents with dual disabilities.

**Method:**

Carers and a young adult with a dual disability were surveyed about their experience of transition care. Other key stakeholders including paediatricians, general practitioners, and policy makers were also interviewed. These data informed the model of care.

**Results:**

Paediatricians and general practitioners reported difficulties establishing working relationships to foster smooth transitions, and carers reported lacking a regular general practitioner with adequate expertise to care for people with dual disabilities. A process of shared care between paediatricians and general practitioners was developed and initiated by a dedicated transition manager, who assisted with care coordination and service linkages. Standardized clinical assessment tools were also introduced to determine patient and carer support needs.

**Interpretation:**

This study highlights the potential to improve transition outcomes for adolescents with dual disabilities and their carers through early transition planning, consistent methods of assessing patient and carer needs, and shared care.

**What this paper adds:**

- Adolescents with co‐occurring disabilities require a collaborative health and disability service interface.
- Fearless, Tearless Transition is a new approach to transitioning adolescents with dual disabilities from paediatric to adult care.
- Carers of adolescents with dual disabilities require support navigating and negotiating services.
- Engaging general practitioners and paediatricians in shared care early during the transition process is essential.

## What this paper adds

- Adolescents with co‐occurring disabilities require a collaborative health and disability service interface.
- Fearless, Tearless Transition is a new approach to transitioning adolescents with dual disabilities from paediatric to adult care.
- Carers of adolescents with dual disabilities require support navigating and negotiating services.
- Engaging general practitioners and paediatricians in shared care early during the transition process is essential.

This article's abstract has been translated into Spanish and Portuguese.

Follow the links from the abstract to view the translations.

This article is commented on by Pettit on page 497 of this issue

Video Podcast: https://youtu.be/Hj1mj2tq4mw

## Desarrollo del modelo de atención de transición sin miedo y sin lágrimas para adolescentes con discapacidad intelectual y / o trastorno del espectro autista con comorbilidades de salud mental

**Objetivo:**

Primero, comprender las barreras para lograr una transición efectiva y los apoyos requeridos desde la perspectiva de los padres y cuidadores, adolescentes con discapacidad intelectual y / o trastorno del espectro autista y trastornos de salud mental coexistentes, a menudo denominados "discapacidad dual", y los que prestan servicios a este grupo. En segundo lugar, desarrollar un modelo informado de atención compartida para mejorar la transición de los adolescentes con discapacidad dual.

**Método:**

Se encuestó a los cuidadores y a un adulto joven con una discapacidad doble sobre su experiencia de cuidados de transición. También se entrevistó a otras partes interesadas clave, incluidos pediatras, médicos generales y responsables políticos. Estos datos informaron el modelo de atención.

**Resultados:**

Los pediatras y los médicos generales informaron sobre las dificultades para establecer relaciones de trabajo para fomentar una transición fluida, y los cuidadores informaron que carecían de un médico general con experiencia adecuada para atender a las personas con doble discapacidad. Un administrador de transición dedicado elaboró ​​e inició un proceso de atención compartida entre pediatras y médicos generales, que ayudó con la coordinación de la atención y los vínculos de servicios. También se introdujeron herramientas de evaluación clínica estandarizadas para determinar las necesidades de apoyo del paciente y el cuidador.

**Interpretación:**

Este estudio destaca el potencial para mejorar los resultados de la transición para los adolescentes con discapacidades duales y sus cuidadores a través de la planificación temprana de la transición, métodos consistentes para evaluar las necesidades del paciente y del cuidador, y la atención compartida.

## Desenvolvimento do modelo de transição do cuidado “Sem medo, sem lágrimas” para adolescentes com deficiência intelectual e/ou transtorno do espectro autista com comorbidades em saúde mental

**Objetivo:**

Primeiro, entender as barreiras para atingir uma efetiva transição, e os suportes necessários da perspectiva dos pais e cuidadores, adolescentes com deficiência intelectual e/ou transtorno do espectro autista e transtornos coexistentes de saúde mental, frequentemente denominados “dupla incapacidade”, e aqueles que oferecem serviços para este grupo. Segundo, desenvolver um modelo informado de cuidado compartilhado para melhorar a transição dos adolescentes com duplas incapacidades.

**Método:**

Cuidadores e um jovem adulto com dupla incapacidade foram entrevistados sobre sua experiência com a transição do cuidado. Outros envolvidos, incluindo pediatras, profissionais generalistas, e tomadores de decisão também foram envolvidos. Estes dados informaram o modelo de cuidado.

**Resultados:**

Pediatras e profissionais generalistas relataram dificuldades em estabelecer relacões de trabalho para estimular transições suaves, e os cuidadores reportaram falta de um professional generalista regular com experiência adequada para cuidar das pessoas com duplas incapacidades. Um processo de cuidado compartilhado foi desenvolvido e iniciado por um gerente dedicado para transição, que auxiliou com a coordenação do cuidado e ligações entre serviços. Instrumentos clínicos padronizados também foram introduzidos para determinar as necessidades de suporte do paciente e cuidador.

**Interpretação:**

Este estudo enfatiza o potencial de melhorar os resultados de transição para adolescentes com duplas incapacidades e seus cuidadores por meio de um planejamento precoce da transição, métodos consistentes de avaliar necesidades de paciente e cuidador, e cuidado compartilhado.

**Abbreviations**
ASD: Autism spectrum disorder
RCH: Royal Children’s Hospital

Transition from paediatric to adult care is challenging for adolescents with developmental disabilities.^1^, ^2^ Young people with intellectual disability and/or autism spectrum disorder (ASD) have higher than average numbers of emergency room visits and non‐emergency medical visits, and a higher incidence of behavioural problems,^3^ as well as difficulties accessing health care^4^ and potentially avoidable deaths.^5^

In Australia, an estimated 164 000 people or 1 in 150 Australians (0.7% prevalence rate) had a diagnosis of ASD in 2015, with 83% of this population under the age of 25 years. This reflected a considerable increase from previous data in 2009, which estimated 64 400 people had a diagnosis of ASD.^6^ A 2016 study suggested that the prevalence of ASD has continued to increase in Australia to 2.5%.^7^ A substantial proportion of children and adolescents with ASD, and/or intellectual disability, have associated severe behavioural problems and mental health disorders, often termed ‘dual disability’.^8^, ^9^

Challenges to the transition of adolescents with dual disabilities relate to the intrinsic complexity of this cohort as well as deficits within health and disability care systems, thus leading to variation in transition planning,^10^ higher levels of anxiety about transitioning,^4^ and, for adolescents with more severe intellectual disability, greater parental reluctance to leave paediatric care compared with young people with chronic illness.^2^ Furthermore, the extremely high burden of care^11^ may lead to issues such as relinquishment of care in addition to acute distress and socio‐economic adversity.^12^ This period of transition has also been associated with the presence of health and well‐being issues, such as obesity and sexual health concerns for people with intellectual disability.^13^ For many, particularly those with severe intellectual disability, transition to adult health care also coincides with concomitant transfers such as the move from school to day placements, and from home to residential care, as well as legal and financial changes.^14^

Deficits in our health (including mental health), social, and disability systems^15^ and the fragmentation of services exacerbate problems during the critical period of transition, and often lead to adolescents and their families feeling confused and disconnected.^2^ Factors contributing to these problems for this population include discontinuity of services,^16^ lack of mental health service involvement in transition planning,^17^ lack of training and capacity of mental health practitioners in managing mental health concerns in this populations,^18^ and resourcing difficulties.^19^

We sought to understand the barriers to achieving effective transition and the supports required from the perspective of parents and carers, people with dual disabilities, and those who provide services, and to develop a model of shared care to improve the transition of young people with dual disabilities.

## METHOD

The development of the Fearless, Tearless Transition model of care was a three‐step process.

### Step one

We consulted with a young adult with a dual disability and carers of young adults with dual disabilities who transferred from the Royal Children’s Hospital (RCH) between 2012 and 2016. We also consulted with other internal and external stakeholders.

Data about the severity of their underlying ASD and/or intellectual disability were collected from clinician notes in the RCH electronic medical record. Participants were invited to complete a survey developed by the project team (Appendix S1, online supporting information). Questions were based on challenges and gaps in transition experienced by adolescents with dual disabilities and their carers identified by completing a literature review, and the expert opinions of the clinical team. Carers and patients with dual disabilities were asked to provide feedback on their experience of transition to adult care, access to and usage of services, and the level of daily care required for their young adult with a dual disability.

Semistructured interviews were conducted with internal and external stakeholders, including general practitioners and mental health practitioners who provide care for people with dual disabilities, and interview questions were codesigned by expert general practitioners from the Centre for Developmental Disability and North Western Melbourne Primary Health Network in collaboration with paediatricians. We purposively identified clinicians to be interviewed based on their experience in looking after this patient group and being nominated by clinicians in the project team. Other expert clinical leads known to the first round of suggested stakeholders were also identified and interviewed within Australia and in other international settings; some were identified after the project team’s presentation at a European adolescent psychiatry conference. Clinicians were asked about their current transition practices, challenges with managing this patient group, and systemic barriers and facilitators for transition.

Semistructured interviews were also conducted with representatives from relevant government agencies and peak bodies informing policies and practice, educators from special development schools, and allied health professionals at RCH and in the community. These external stakeholders were identified by RCH clinicians as having experience with adolescents and young adults with dual disabilities across various roles. Data were also collected about primary roles and locations of practice. Data were analysed thematically using an inductive approach.

### Step two

After feedback from stakeholders, a new model of care was developed and is described. The model was then implemented with paediatricians and the Transition Support Service at RCH and local treating general practitioners.

### Step three

During implementation of the model, information was gathered from carers, paediatricians, and general practitioners participating in the model (manuscript in preparation). A general practitioner engagement strategy was developed and clinical assessment tools were identified and adapted for clinical use.

### Ethical approval

Approval from a human research ethics committee was not sought for steps one and two of this project as it was deemed to be a quality improvement activity and did not require ethical review. The project steering committee provided ethical oversight of the project. Participation in the project was voluntary and all participants provided informed written consent. The RCH human research ethics committee approved this project before step three and implementation of the model (HREC#37341).

## RESULTS

### Step one: understanding barriers and enablers to effective transition

#### Participants

Both young adults and their carers were invited to complete the survey. A total of 19 people responded to the survey; 18 carers and one young adult. Fourteen of the carers reported that their young adults had diagnoses of both intellectual disability and ASD, two carers had young adults with intellectual disability, and two carers had young adults with ASD. One young adult reported she had both intellectual disability and ASD. Of the two young people with intellectual disability alone, their reported intellectual disability was moderate (one) and severe (one). Of the 15 young people with both intellectual disability and ASD, their reported intellectual disability was mild (four), moderate (three), and severe (eight). Of the two young people with ASD alone, their reported ASD was mild (one) and moderate (one). Of the 15 young people with both intellectual disability and ASD, their reported ASD was mild (two), moderate (three), and severe (10). Carers reported their young adults had the following associated mental health conditions: anxiety disorder (12), attention‐deficit/hyperactivity disorder (five), and behaviour of concern including severe aggression (11), depression (four), and bipolar disorder (one). The young adult reported their associated mental health condition to be schizophrenia.

A total of 62 internal and external stakeholders were also interviewed, including two general practitioners with extensive clinical experience in the management of patients with dual disabilities and a wider advocacy and educational role within larger networks of general practitioners. The characteristics of stakeholders are described in Table 1.

**Table 1:** Stakeholder demographics

Characteristics	RCH staff	External professionals
*Role*		
Paediatrician	17	10
Neurologist	2	1
Psychiatrist	2	9
Psychologist	1	1
Rehabilitation specialist	1	1
Gynaecologist	1	0
General practitioner	0	2
Clinical nurse consultant	1	0
Allied health	1	1
Advocacy worker	0	1
Academic	0	3
Teacher	0	2
Government	0	5
*Location*		
Victoria, Australia	26	23
New South Wales, Australia	0	4
Switzerland	0	5
England	0	1
Italy	0	1
The Netherlands	0	1
Germany	0	1

RCH, Royal Children’s Hospital.

#### Major concerns and unmet need themes

Overall, the key concerns and identified needs that informed the model were as follows (see Table S1, online supporting information, for examples of illustrative quotes).

##### Variation in transition planning and management

Clinicians, policy makers, and educators perceived variability in planning for the transition of patients with dual disabilities in the health, disability, and community settings as a challenge. Many cited time constraints, resource issues, and a lack of coordination as contributing to this variation in practice. Centralized case management, such as a dedicated transition coordinator role, was perceived to aid successful transition of patients with dual disabilities by clinicians and carers. Many reported the transition process as requiring an early start, a team approach, and structured planning with clear patient‐ and family‐centred communication. Assisting families to view transition as a continuous journey with good preparation and providing support in navigating adult care was regarded as an important part of the transition process for both carers and clinicians.

##### Systemic disconnection between health and disability

The disconnection between the health and disability sectors was perceived as a barrier, with flawed communication systems between essential services; an issue compounded by inherent differences between paediatric and adult health systems, and between health and disability. Clinicians reported experiencing frustrations when trying to find the right service and referring to adult health and disability services. Young people with dual disabilities and their carers all reported limited to no communication within and between service providers, within and across sectors.

##### Lack of services and expertise

A lack of available and/or appropriate services in the community including general practice, psychiatry, psychology, allied health, and disability supports, particularly for moderate to severe levels of disability, was consistently reported by clinicians and carers. Carers frequently cited that their health care providers, especially general practitioners, were not experienced in dealing with people with autism and other developmental disabilities. General practitioners also reported limited knowledge in managing people with dual disabilities, especially with regard to psychotropic medications. The majority of carers reported they had a regular general practitioner, yet they were not helpful during their transition. The lack of expertise and training in the management of patients with dual disabilities in psychiatry, general practice, and adult health services was raised as a key concern amongst clinicians. Carers also reported their young adults had high supervision and daily care needs, which further contributed to difficulties in finding appropriate services.

##### Carer anxiety regarding transition

Lack of family and patient preparedness to transfer, resulting in high levels of carer anxiety, was a common issue. Carers and clinicians often cited the differences between paediatric and adult care as contributing to high levels of carer anxiety. Inherent differences between paediatric and adult services were commonly cited as a barrier amongst all clinicians, and adult clinicians noted there were unrealistic expectations of adult services, for example, access to a consistent clinician at each visit, differences in resourcing, and often increased clinical demands within the adult health system.

##### Dissatisfaction and unmet needs during transition to adult care

Carers consistently reported their child’s medical, social, vocational, developmental, and sexuality needs were not met to their satisfaction during the transition period. Overall, there was high dissatisfaction with their medical transition to adult services. Some carers felt a sense of abandonment and disappointment after their child’s care was transferred to adult services, particularly in the mental health system.

##### Building capacity, collaboration, and shared care

Providing specialized training opportunities to upskill and empower mental health practitioners and general practitioners to care for young adults with dual disabilities was noted as a primary area of importance, along with the need to develop an expert network. The importance of developing partnerships, strong communication, and shared care between hospital practitioners and general practitioners was highlighted, with the role of the general practitioner raised as key in enabling good continuity of care.

### Step two: model development and description

The data collected from step one were used to inform a model of transition care, ‘Fearless, Tearless Transition’, in the following ways. First, to improve carer satisfaction, reduce anxiety regarding transition, and to reduce variation in planning, a centralized family‐centred transition support model was employed early with an expert transition manager coordinating and enabling greater connectivity to appropriate services for patients and their families, and providing a single point of contact for carers. Further to this, the use of consistent clinical assessment tools to identify patient and carer support needs early, and the partnerships established between paediatricians and general practitioners, aimed to build carer confidence in care provided within the community. Second, to address the disconnection between health and disability, and to build capacity and increase expertise in the management of patients with dual disabilities, the project team established relationships with community health professionals, and engaged in advocacy and educational activities to build knowledge. This included the shared care paediatrician and general practitioner process, the implementation of the general practitioner Health Pathway, establishment of partnerships with adult health and disability services, and sharing of knowledge at clinical forums. Table S2 (online supporting information) describes the key stages of the model.

### Step three: model implementation

#### General practitioner engagement strategy

The project paediatrician led the general practitioner engagement strategy during the first year of the implementation process and was instrumental in providing direct communications with general practitioners, helping to build capacity in general practice through promotion of the shared care paediatrician and general practitioner process and the development of the general practitioner Health Pathway (https://melbourne.healthways.org.au/), a free web portal with information for general practitioners on the management of patients with dual disabilities. The Health Pathway was developed in partnership with a general practitioner clinical editor, a panel of expert paediatricians and general practitioners in the community. The RCH dual disability psychiatrist (CP) was involved with identifying and expanding the network of private psychiatrists in the community with expertise in managing adults with dual disabilities.

#### Clinical assessment tools

The Health of the Nations Outcomes Scale – Learning Disability,^20^ Autism Parenting Stress Index,^21^ and a modified Supervision Rating Scale^22^ were used by paediatricians to assess mental health symptoms and behaviours of concern, parental stress, and carer burden and the level of supervision required respectively (Table S3 and Appendices [Link], [Link], [Link], online supporting information). These tools, initially used in the RCH Dual Disability clinic, were proposed for this project by the dual disability psychiatrist in consultation with the paediatricians in our project team, and subsequently adapted and standardized for clinical use by senior clinicians. These assessments were administered at each of the three key stages described in the model (Table S2), with the addition of the 12‐year‐old checklist (Appendix S5, online supporting information) administered at the first stage to assess early transition needs.

After completion of clinical assessment tools, paediatricians, the psychiatrist, and/or the transition manager engaged relevant supports for patients and their carers. Structured information was collected at three time points, and routine clinical and transition care appointments allowed more frequent contact and communication with families to ensure identified needs were followed up, including after transfer to ensure successful engagement with adult care. This process of follow‐up throughout the transition and transfer period will enable continued learnings and improvements to benefit families, and contribute to greater knowledge for clinicians caring for them.

## DISCUSSION

Transition for an adolescent with a dual disability is often managed ad hoc. Common challenges that occur for each transition are that the paediatrician role is not usually represented in adult health services, and so there is increased reliance on general practitioners to assume care. Unlike other health settings, such as the UK where patients register with specific general practitioners and maintain this relationship, Australia has a less‐structured system whereby patients may have difficulties finding a regular general practitioner, resulting in multiple or no general practitioners when leaving paediatric care. Furthermore, adult health care providers may be engaged at multiple sites and care may be decentralized, and public psychiatry services for this cohort are scarce resulting in high costs for families who have no option but to engage with private psychiatrists for the management and monitoring of psychotropic medications. Effective transition planning for adolescents with dual disabilities requires a holistic, collaborative, and family‐centred approach that considers the support needs of both the young person and the carer.^1^, ^2^

Our scoping results identified several opportunities for improvement, including the need for dedicated transition appointments, essential in reducing the variation in transition care and providing vital information and support, for carers navigating and negotiating adult health and community services. This finding is consistent with other research on the health care transition requirements of young people with autism.^23^ Carers experience high burden of care and difficulties navigating and accessing services at a time when there is a greater need for support in managing the vocational, educational, sexual health, independent living, guardianship, and other needs of their adolescents and young adults.^1^, ^2^ Centralized models of care coordination are associated with a reduction in unmet health care needs, improvements in youth functional status,^24^ reduction in health care costs, reductions in barriers to care, and greater patient and family satisfaction.^25^

While general practitioners often assume management of adults with dual disabilities, they may have minimal involvement during paediatric years leading to a deskilling of the workforce and exacerbation of an ineffective family and general practitioner connection. The shared care component of this model seeks to address this issue through early engagement of general practitioners jointly with paediatricians to care for adolescents with dual disabilities. This enables more time for general practitioners to develop an understanding of the adolescent’s and family's needs while also building trust. In addition to earlier engagement, resources developed in this project, including the general practitioner Health Pathway and the shared care communication brochure, are a means of upskilling the general practitioner workforce.

The mental health needs of young people aged 19 to 24 are also more likely to require appropriate interventions and care, as compared with those aged 13 to 18 years,^26^ and for patients with intellectual disability and/or ASD, mental health concerns such as anxiety disorders are present in between 22% to 84% of the population.^27^ These co‐existing mental health concerns, such as the development of anxiety disorders, warrant additional focus and transition support to ensure that a stressful transition period is managed well.

The Fearless, Tearless Transition model screens for mental health concerns through the use of Health of the Nation Outcome Scale – Learning Disabilities at key stages of adolescence, providing opportunities to review treatments and to implement appropriate interventions and supports as required well before transfer of care. Although evaluation of this model is yet to be published, early results indicate carers who received this intervention reported feeling more prepared for transition and were more regularly engaged with their general practitioners. This model has also been adopted by clinicians in the community with regular consultations provided by the transition manager and clinicians in the project team, suggesting increased recognition of the potential for this model to improve transition outcomes for young people and their families.

### Sustainability

To ensure the sustainability of Fearless, Tearless Transition, clinical assessment tools and resources are integrated into the hospital’s electronic database. Moreover, opportunities to build further capacity at RCH and in the community continue to be fostered through the shared care process with general practitioners and across various other professional forums. Patients with dual disabilities and their carers continue to be supported in RCH transition clinics with the transition manager to assist with service linkages and care coordination. Further afield, this model and its resources has been shared with other health settings across Australia and with some interested international groups, including Evelina Children’s Hospital in London, paving the way for potential cross‐evaluation collaborations in future to refine and evaluate the model in different health systems.

### Limitations

As Fearless, Tearless Transition was developed with the existence of a well‐established, centralized, hospital‐wide transition service for carers, the replicability of an equivalent transition support process within other settings may be challenging; however, many aspects of this model can be easily translated into other health settings. This includes the adoption of the clinical assessment tools, resources, the family‐centred approach, shared care between paediatricians and general practitioners, and capacity‐building work.

Another limitation was the participation of only one young adult with a dual disability, despite inviting both carers and young adults to complete the survey in step one. Including the perspectives of young adults with dual disabilities is needed in future transition research.

## CONCLUSION

This study identified that variation in transition planning and management, systemic disconnection between health and disability, lack of services and expertise, high levels of carer anxiety regarding transition, high levels of dissatisfaction, and unmet needs during transition to adult care are barriers to providing optimal transition from paediatric to adult care for patients with dual disabilities. To address these barriers, we developed the Fearless, Tearless Transition model to build capacity in the workforce, through collaboration and shared care between paediatricians and general practitioners. Our model highlights the potential to improve transition outcomes for adolescents with dual disabilities and their carers by providing a structured, centralized, and family‐centred transition process. This model of care has generated interest at the national and international level within different health jurisdictions, and learnings from this project may be translated into other settings. Funding bodies and policy makers should be encouraged to dedicate targeted resources to assist in the future development of interventions such as Fearless, Tearless Transition to support vulnerable adolescents with dual disabilities and their carers as they transition from paediatric to adult care.

## Supporting information


**Table S1**: Key themes and illustrative quotes

**Table S2**: Key stages of the Fearless, Tearless Transition model

**Table S3**: Clinical assessment descriptions

**Appendix S1:** Questionnaire for carers and young adults.

**Appendix S2:** Modified Health of the Nations Outcomes Scale – Learning Disability and scoresheet.

**Appendix S3:** Autism Parenting Stress Index.

**Appendix S4:** Modified Supervision Rating Scale.

**Appendix S5:** 12‐year‐old checklist.

## Data Availability

De‐identifiable data that support the findings of this study are available from the corresponding author upon reasonable request.
