# Supplementary material for: Development of the Fearless, Tearless Transition model of care for adolescents with an intellectual disability and/or autism spectrum disorder with mental health comorbidities
Source: Dev Med Child Neurol. 2020 Dec 17;63(5):560–5. doi: 10.1111/dmcn.14766 (PMC8247054; doi:10.1111/dmcn.14766)
Supplement: Supplementary file 1 — Table S1: Key themes and illustrative quotes [file DMCN-63-560-s001.docx]

**Table S1**: Key themes and illustrative quotes

| Key theme | Illustrative quotes |
| --- | --- |
| Variation in transition planning and management | ‘No systematic approach (to transition)’ (psychiatrist, RCH) |
|  | ‘Individual case management and advocacy is key’ (paediatrician, RCH) |
|  | ‘Being encouraged to think about it before he turned 18, so he could process it in his thinking and be ready to move across earlier’ (carer) |
| Systemic disconnection between health and disability | ‘Health and communication services/disability sectors working independently’ (paediatrician, community) |
| Lack of services and expertise | ‘Should he get ill, we only have a GP who has no knowledge of autism or complex needs’ (carer) |
|  | ‘The lack of availability and accessibility of adult services providers with skills in ID and ASD presents challenges for transition’ (paediatrician, RCH)  ‘He needs two-to-one support for all his daily needs and structure, and constant supervision to remain safe, calm, and to deal with aggressive outbursts’ (carer)  ‘Inadequate supports lead to relinquishing the patient as parents become exhausted and can’t cope’ (paediatrician, community) |
| Carer anxiety regarding transition | ‘The patients find the adult world daunting and an environment that is inappropriate to manage their problems, even something as simple as the waiting room’ (paediatrician, RCH) |
|  | ‘It's quite a challenge. It took me 5 years to get used to the transition and it’s quite an emotional experience’ (carer) |
|  | ‘Paeds create an expectation in the adult world that can’t be matched’ (paediatrician, community) |
| Dissatisfaction and unmet needs during transition to adult care | ‘My autism was severe and I had to deal with a lot of hurdles and I did not get the support. I had to face challenges all on my own’ (young adult)  ‘It has just been a nightmare and one that hasn’t gone away. Transition was very painful for all of us’ (carer)  ‘The paediatrician attempted to set up supports, but I have heard nothing so we definitely feel dumped’ (carer)  ‘I was so appalled (at the mental health service), it was the worst joke I have ever experienced’ (carer) |
| Building capacity, collaboration and shared care | ‘Understanding comorbid MH issues… need a specialist physician to take on care with appropriate training’ (paediatrician, RCH) |
|  | ‘We need much stronger partnerships with GPs and to empower them to do what we do’ (paediatrician, RCH) |

RCH, Royal Children’s Hospital; GP, general practitioner; ID, intellectual disability; ASD, autism spectrum disorder; MH, mental health.
