# Supplementary material for: Development of the Fearless, Tearless Transition model of care for adolescents with an intellectual disability and/or autism spectrum disorder with mental health comorbidities
Source: Dev Med Child Neurol. 2020 Dec 17;63(5):560–5. doi: 10.1111/dmcn.14766 (PMC8247054; doi:10.1111/dmcn.14766)
Supplement: Supplementary file 2 — Table S2: Key stages of the Fearless, Tearless Transition model [file DMCN-63-560-s003.docx]

**Table S2**: Key stages of the Fearless, Tearless Transition model

| Stage one (patients aged 12–14y) |
| --- |
| - Patients are identified by paediatricians as appropriate to commence the Fearless, Tearless Transition model - Paediatricians complete the 12-year-old checklist and clinical assessment tools (see Table 4) to assist with identifying care needs and review at subsequent appointments - Paediatricians also ask families what is important to them for their child and set goals with the family - Paediatrician recommends supports based on family needs and goals - Early discussions about transition are initiated between the paediatrician and family |
| Stage two (patients aged 15–17y) |
| - Patients are identified by paediatricians as appropriate for the Fearless, Tearless Transition model - Paediatricians complete the clinical assessment tools to assist with identifying care needs - Areas of concerns from previous checklists and assessments are reviewed - Paediatricians refer to RCH transition clinic - Carers and patients are seen in the transition clinic by a dedicated transition manager, where active transition planning is commenced including care coordination and initiation of important support services, and links to assist with current and future needs - The family is connected with a regular GP if necessary - A shared care process commences between the adolescent's paediatrician and the regular GP - The Fearless, Tearless Transition parent brochure is used to facilitate shared care |
| Stage three (patients aged 18y) |
| - Patients are identified by paediatricians as appropriate for the Fearless, Tearless Transition model - Paediatricians complete the clinical assessment tools to assist with identifying care needs - Areas of concerns from previous checklists and assessments are reviewed - Shared care process between paediatricians and GPs continues until the transfer - Final reviews with paediatrician and transition manager - Transition manager provides additional assistance with care coordination and support linkages where required - Patient is discharged from paediatric care and transfers to adult care |

RCH, Royal Children’s Hospital; GP, general practitioner.
