# Supplementary material for: Development of the Fearless, Tearless Transition model of care for adolescents with an intellectual disability and/or autism spectrum disorder with mental health comorbidities
Source: Dev Med Child Neurol. 2020 Dec 17;63(5):560–5. doi: 10.1111/dmcn.14766 (PMC8247054; doi:10.1111/dmcn.14766)
Supplement: Supplementary file 3 — Table S3: Clinical assessment descriptions [file DMCN-63-560-s007.docx]

**Table S3**: Clinical assessment descriptions

| Clinical assessment tool | Description |
| --- | --- |
| M-HoNOS-LD^20^ | The M-HoNOS-LD is used by paediatricians to assess the range and severity of MH symptoms and behaviours of concern in adolescents. Scores achieved on the HoNOS-LD are correlated with recommended community supports with higher scores indicating increased severity and support required. The HoNOS-LD was developed and validated for use with young adults with mild to borderline ID, and severe behaviour and mental health problems, and was found to have fair to good internal consistency and intraclass coefficients for (sub)scales ranging from fair to good.^28^ This was modified from 18 to 10 items by the RCH dual disability psychiatrist and selected as the tool of choice by paediatricians (see Supporting File 1, online supporting information). A video was also developed to assist paediatricians with the use of the M-HoNOS-LD. |
| APSI^21^ | The APSI is a 13-item scale used to measure parental stress and carer burden in managing challenging behaviours and other aspects of the disability, and their concerns for the future (see Supporting File 2). This scale is rated from ‘not stressful’ to ‘so stressful we feel we cannot cope’, with demonstrated acceptable internal consistency and test–retest reliability for parents of children with developmental difficulties. |
| M-SRS^22^ | The M-SRS is used to measure the level of supervision required on a 13-point ordinal scale that can be optionally grouped into five ranked categories (independent, overnight supervision, part-time supervision, full-time indirect supervision, and full-time direct supervision). This clinical assessment tool was modified very minimally by adding a few explanatory words to some item headings to assist the clinician’s understanding of the required ratings. None of the items themselves on the original SRS were modified or changed in any way (see Supporting File 3). |
| 12-year-old checklist | A 12-year-old checklist (see Supporting File 4) was also developed by the project team to assist paediatricians in assessing the support needs and services required by patients with dual disabilities, and their carers, in stage one of the model. |

M-HoNOS-LD, Modified Health of the Nations Outcomes Scale – Learning Disability; MH, mental health; HoNOS-LD, Health of the Nations Outcomes Scale – Learning Disability; ID, intellectual disability; RCH, Royal Children’s Hospital; APSI, Autism Parenting Stress Index; M-SRS, Modified Supervision Rating Scale; SRS, Supervision Rating Scale.
