# Supplementary material for: Development of the Fearless, Tearless Transition model of care for adolescents with an intellectual disability and/or autism spectrum disorder with mental health comorbidities
Source: Dev Med Child Neurol. 2020 Dec 17;63(5):560–5. doi: 10.1111/dmcn.14766 (PMC8247054; doi:10.1111/dmcn.14766)
Supplement: Supplementary file 4 — Appendix S1: Questionnaire for carers and young adults. [file DMCN-63-560-s002.pdf]

QUESTIONNAIRE FOR CARERS AND YOUNG ADULTS

Intellectual Disability and/or Autism Spectrum Disorder and Mental Health Transition

Name (Parent / Carer or Patient) – *Please highlight*		Date	
Child's Name		Relationship	
Email		Phone	

BACKGROUND INFORMATION

a. What diagnoses have you/your child been given? ***Please circle or highlight***

- ☐ Intellectual Disability – MILD MODERATE SEVERE
- ☐ Autism Spectrum Disorder – MILD MODERATE SEVERE
- ☐ Anxiety – MILD MODERATE SEVERE
- ☐ Attention Deficit Hyperactivity Disorder – MILD MODERATE SEVERE
- ☐ Depression – MILD MODERATE SEVERE
- ☐ Major behaviour disorder – MILD MODERATE SEVERE
- ☐ Other Mental Health Diagnoses – Please List
  - i. ______________________ – MILD MODERATE SEVERE
  - ii. ______________________ – MILD MODERATE SEVERE

b. What are some of the challenges you currently face with your/your child's physical and mental health and their daily care needs?

c. How old are you/your child and at what age did you/your child transfer from paediatric care?

d. Did you feel this was a good age to transfer? Why?

TRANSITION PLANNING

a. At what age was transition to adult care first discussed with you/your child?

b. Was your GP helpful during transition? (Please provide details)

c. If you were not engaged with a GP, please provide details.

d. How do you rate your experience of transition in the areas below (1 = very dissatisfied – 5 = very satisfied)?

- ☐ Medical care/needs - 1 2 3 4 5
- ☐ Social needs - 1 2 3 4 5
- ☐ Vocational needs - 1 2 3 4 5
- ☐ Developmental needs - 1 2 3 4 5
- ☐ Sexuality needs - 1 2 3 4 5

e. Were there any challenges or unexpected issues that you experienced during transition?

f. Are there any changes you would suggest to improve the transition process?

FROM TRANSITION AND BEYOND

a. What services do you/your child currently use (please provide details)?

   a. Medical: ____

   b. Mental health:____

   c. Vocational:____

   d. Respite:____

   e. Allied health: :____

   f. Other : ____

b. Were there services you wanted to engage with but were not able to access? (if yes, please list)

c. Did the transition process give accurate information on what to expect in adult services?

d. Did you/your child feel included in the transition process?

e. Once transferred to adult services, did you/your child experience any issues? (if yes, please provide details)

f. What differences have you noticed in the care provided in adult services?

   i. Medical -
   ii. Vocational -
   iii. Social -
   iv. Mental health –

v. Other -

g. What resources or services stand out as being beneficial for you/your child?

h. If applicable, how would you describe the impact of changing from child to adult mental health services?

i. Did the transition process assist you in developing skills or knowledge in the following areas? (Yes or No)

Topics of discussion	Carer	Young adult
Communicating with professionals		
Decision making, self-advocacy and independence		
Self-care		
Activities of daily living		
Safety		
Socialisation		
Transportation		
Legal and financial issues		
Work		
Education		
Navigating the health system		
Managing medications and other medical information		

j. Is there anything you think we should know about your transition experience which may be helpful to others?

Thank you for your time today as your feedback is extremely helpful. If you have any further comments or questions in relation to this survey, please contact:
