# Supplementary material for: Development of the Fearless, Tearless Transition model of care for adolescents with an intellectual disability and/or autism spectrum disorder with mental health comorbidities
Source: Dev Med Child Neurol. 2020 Dec 17;63(5):560–5. doi: 10.1111/dmcn.14766 (PMC8247054; doi:10.1111/dmcn.14766)
Supplement: Supplementary file 5 — Appendix S2: Modified Health of the Nations Outcomes Scale – Learning Disability and scoresheet. [file DMCN-63-560-s006.docx]

**ROYAL CHILDREN’S HOSPITAL ASD/ID TRANSITION PROJECT**

**HoNOS-LD MENTAL HEALTH SPECIFIC ITEMS**

Summary rating instructions:

1. Rate each in order from item 1 to 10.
2. Do not include information rated in an earlier item.
3. Rate the person over the previous 4 weeks.
4. Rate the most severe problem that has occurred during the period rated.
5. All items follow the five-point rating format similar to other HoNOS instruments:
   - 0=no problem during the period rated;
   - 1=mild problem;
   - 2=moderate problem;
   - 3=severe problem;
   - 4=very severe problem.

**Rate 9 if unknown**

**1. Behavioural problems (directed at others)**

**Include** behaviour that is directed to other persons. **Do not include** behaviour that is directed towards self (Scale 2) or primarily at property or other behaviours (Scale 3). Rate risk as it is currently perceived.

- 0= No behavioural problems directed to others during the period rated.
- 1= Irritable, quarrelsome, occasional verbal abuse.
- 2= Frequent verbal abuse, verbal threats, occasional aggressive gestures, pushing or pestering (harassment).
- 3= Risk, or occurrence of, physical aggression resulting in injury to others requiring simple first aid, or requiring close monitoring for prevention.
- 4= Risk, or occurrence of, physical aggression producing injury to others serious enough to need casualty treatment and requiring constant supervision or physical intervention for prevention (e.g. restraint, medication or removal).

### 2. Behavioural problems directed towards self (self-injury)

**Include** all forms of self-injurious behaviour. **Do not include** behaviour directed towards others (Scale 1), or behaviour primarily directed at property, or other behaviours (Scale 3).

- 0= No self-injurious behaviour during the period rated.
- 1= Occasional self-injurious behaviour (e.g. face-tapping); occasional fleeting thoughts of suicide.
- 2= Frequent self-injurious behaviour not resulting in tissue damage (e.g. redness, soreness, wrist-scratching).
- 3= Risk or occurrence of self-injurious behaviour resulting in reversible tissue damage and no loss of function (e.g. cuts, bruises, hair loss).
- 4= Risk or occurrence of self-injurious behaviour resulting in irreversible tissue damage and permanent loss of functions (e.g. limb contractures, impairment of vision, permanent facial scarring) or attempted suicide.

### 3. Other mental and behavioural problems

This is a global rating to include behavioural problems not described in Scales 1 or 2. **Do not include** behaviour directed towards others (Scale 1), or self-injurious behaviour (Scale 2). Rate the most prominent behaviours present. **Include:** A, behaviour destructive to property; B, problems with personal behaviours, for example, spitting, smearing, eating rubbish, self-induced vomiting, continuous eating or drinking, hoarding rubbish, inappropriate sexual behaviour; C, rocking, stereotyped and ritualistic behaviour; D, anxiety, phobias, obsessive or compulsive behaviour; E, others.

- 0= No behavioural problem(s) during the period rated.
- 1= Occasional behavioural problem(s) that are out of the ordinary or socially unacceptable.
- 2= Behaviour(s) sufficiently frequent and severe to produce some disruption of and impact on own or other people's functioning.
- 3= Behaviour(s) sufficiently frequent and severe to produce significant disruption and impact on own or other people's functioning, requiring close monitoring for prevention.
- 4= Constant, severe problem behaviour(s) producing major disruption of and impact on functioning requiring constant supervision or physical intervention for prevention.

### 4. Attention and concentration

**Include** problems that may arise from underactivity, overactive behaviour, restlessness, fidgeting or inattention, hyperkinesis or arising from drugs.

- 0= Can sustain attention and concentration in activities/programmes independently during the period rated.
- 1= Can sustain attention and concentration in activities/programmes with occasional prompting and supervision.
- 2= Can sustain attention and concentration in activities/programmes with regular prompting and supervision.
- 3= Can sustain attention and concentration in activities/programmes briefly with constant prompting and supervision.
- 4= Cannot participate in activities and programmes even with constant prompting and supervision.

### 5. Memory and orientation

**Include** recent memory loss and worsening of orientation for time, place and person in addition to previous difficulties.

- 0= Can reliably find their way around familiar surroundings and relate to familiar people.
- 1= Mostly familiar with environment/person, but with some difficulty in finding their way.
- 2= Can relate to environment/person with occasional support and supervision
- 3= Can relate to environment/person with regular support and supervision.
- 4= Not apparently able to recognise or relate to people and environments

### 6. Problems associated with hallucinations and delusions

**Include** hallucinations and delusions irrespective of diagnosis. **Include** all manifestations suggestive of hallucinations and delusions (responding to abnormal experiences, e.g. invisible voices when alone).

- 0= No evidence of hallucinations or delusions during period rated.
- 1= Occasional odd or eccentric beliefs or behaviours suggestive of hallucinations or delusions.
- 2= Manifestations of hallucinations or delusions with some distress or disturbance.
- 3= Manifestations of hallucinations or delusions with significant distress or disturbance.
- 4= Mental state and behaviour are seriously and adversely affected by hallucinations or delusions with severe distress or disturbance.

### 7. Problems associated with mood changes

**Include** problems associated with low mood states, elated mood states, mixed moods and mood swings (alternating between unhappiness, weeping and withdrawal on one hand and excitability and irritability on the other).

- 0= No evidence of mood change during period rated.
- 1= Mood present but with little impact (e.g. gloom).
- 2= Mood change producing significant impact on self or others (e.g. weeping spells, decrease in skills, withdrawal and loss of interest).
- 3= Mood change producing major impact on self or others (e.g. severe apathy and unresponsiveness, severe agitation and restlessness).
- 4= Depression, hypomania or mood swings producing severe impact on self and others (e.g. severe weight loss from anorexia or overactivity, agitation too severe to allow time to be engaged in meaningful activity).

### 8. Problems with sleeping

Do not rate intensity of behaviour disturbance — this should be included in Scale 3. **Include** daytime drowsiness, duration of sleep, frequency of waking and diurnal variation of sleep pattern.

- 0= No problem during the period rated.
- 1= Occasional mild sleep disturbance with occasional waking.
- 2= Moderate sleep disturbance with frequent waking, or some daytime drowsiness.
- 3= Severe sleep disturbance or marked daytime drowsiness (e.g. restlessness/overactivity/waking early) on some nights.
- 4= Very severe sleep disturbance with disturbed behaviour (e.g. restlessness/overactivity/waking early most nights).

### 9. Problems with eating and drinking

**Include** both increase and decrease in weight. Do not rate pica - which should be rated in Scale 3. This scale does not include problems experienced by people who cannot feed themselves (e.g. people with severe physical disability).

- 0= No problem with appetite during the period rated.
- 1= Slight alteration to appetite.
- 2= Severe alteration in appetite with no significant weight change.
- 3= Severe disturbance with some weight change during the period rated.
- 4= Very severe disturbance with significant weight change during the period rated.

### 10. Problems with relationships

**Include** effects of problems with relationships with family, friends and carers (in residential and day/leisure settings). Measure what is occurring regardless of cause, for example, somebody who is known to have good relationships may still display problems.

- 0= Positive and frequent contact with family or friend or carers.
- 1= Generally positive relationships, but some strain or limitations in contact.
- 2= Some positive relationships, but current disruptions of contact or worsening of relationships.
- 3= Difficulties in relationships with risk of breakdown or infrequent contact.
- 4= Significant relationships broken down with no current contact.

**HoNOS-LD score sheet**

**Date Completed: _____________________________________________________**

**Name of person completing HoNOS-LD: ___________________________________**

***Scale 0-4***

***Rate 9 if not known***

1. Behavioural problems (directed at others)
2. Behavioural problems directed towards self (self-injury)
3. Other mental and behavioural problems (specify problem A, B, C, D or E)
4. Attention and concentration
5. Memory and Orientation
6. Problems associated with hallucinations and delusions
7. Problems associated with mood changes
8. Problems with sleeping
9. Problems with eating and drinking
10. Problems with relationships

Total Score

Subjective Score

*Note: All patients should be engaged with DHHS Disability or NDIS*

| **HONOS-LD Score** | **Follow up arrangements required** |
| --- | --- |
| **Below 10**   - **GP** - **DHHS Disability / NDIS** | 1. GP follow up 2. Annual review with Paediatrician alternating with GP i.e. will see a doctor once every 6 months through transition. 3. Prior to transfer all documentation given to GP with a letter and phone call. |
| **Between 10 - 18**   - **GP** - **Psychologist** - **DHHS Disability / NDIS** | 1. GP follow up 2. Annual review with Paediatrician alternating with GP 3. Prior to transfer all documentation given to GP with a letter and phone call. 4. Referral to psychologist from GP (APTAS/Mental Health Care Plan) |
| **Between 18 - 33**   - **GP** - **Psychologist** - **Psychiatrist** - **DHHS Disability / NDIS** | 1. GP follow up 2. Bi-annual review with Paediatrician alternating with GP i.e. will see a doctor once every 3 months through transition. 3. Prior to transfer all documentation given to GP with a letter and phone call. 4. Referral to private psychiatrist including transfer documentation 5. Referral to psychologist from GP (APTAS/Mental Health Care Plan) |
| **Between 33 - 40**   - **GP** - **Adult public MHS +/- Adult Dual Disability Services** - **DHHS Disability / NDIS** | 1. GP follow up 2. Bi-annual review with Paediatrician alternating with GP 3. Prior to transfer all documentation given to GP with a letter and phone call. 4. Referral to public adult MHS +/- adult dual disability service with transfer documentation, plan and meeting if required. |
