# Supplementary material for: Development of the Fearless, Tearless Transition model of care for adolescents with an intellectual disability and/or autism spectrum disorder with mental health comorbidities
Source: Dev Med Child Neurol. 2020 Dec 17;63(5):560–5. doi: 10.1111/dmcn.14766 (PMC8247054; doi:10.1111/dmcn.14766)
Supplement: Supplementary file 7 — Appendix S4: Modified Supervision Rating Scale. [file DMCN-63-560-s005.docx]

**MODIFIED LEVEL OF CARE AND SUPERVISION RATING SCALE (SRS)**

**Name:**

**DOB: Date of rating: Rater:**

| 1 | **Level 1: INDEPENDENT/MINIMAL SUPERVISION**  The patient lives alone or independently. Other persons can live with the patient, but they do not take responsibility for supervision (for example, a child or elderly person). |
| --- | --- |
| 2 | The patient is unsupervised overnight. The patient lives with one or more persons who *could* be responsible for supervision during the day time (for example, a carer, spouse or roommate), but they are *all* sometimes absent overnight. |
| 3 | **Level 2: OVERNIGHT SUPERVISION ONLY**  The patient is only supervised overnight. One or more supervising persons are always present overnight but they are *all* sometimes absent for the rest of the day. |
| 4 | **Level 3: PART-TIME SUPERVISION** The patient is supervised overnight and part-time during waking hours, but is allowed out to attend school/TAFE etc. or on independent outings. One or more supervising persons are always present overnight and are also present during part of waking hours every day. However, the patient is sometimes allowed to leave the residence without being accompanied by someone who is responsible for supervision. |
| 5 | The patient is supervised overnight and part-time during waking hours, but is unsupervised during working hours. Supervising persons are *all* sometimes absent for enough time for them to study &/or work full-time outside the home. |
| 6 | The patient is supervised overnight and during most waking hours. Supervising persons are *all* sometimes absent for periods longer than one hour, but less than the time needed to study/hold a full-time job away from home. |
| 7 | The patient is supervised overnight and during almost all waking hours. Supervising persons are *all* sometimes absent for periods shorter than one hour. |
| 8 | **Level 4: FULL-TIME INDIRECT SUPERVISION** The patient is under full-time indirect supervision. At least one supervising person is *always* present, but the supervising person does not check on the patient more than once every 30 minutes. |
| 9 | Same as #8 plus requires overnight safety precautions (for example, a deadbolt on outside door). |
| 10 | **Level 5: FULL-TIME DIRECT SUPERVISION** The patient is under full-time direct supervision. At least one supervising person is always present and the supervising person checks on the patient more than once every thirty minutes. |
| 11 | The patient lives in a setting in which the exits are physically controlled by others (for example, a locked ward or community residential placement). |
| 12 | Same as #11 plus a supervising person is designated to provide full-time line-of-sight supervision (for example, an escape watch or suicide watch). |
| 13 | The patient is in physical restraints. |

**Circle the rating closest to the level of supervision by family or carers that the person actually receives. ‘Supervision’ means that someone is responsible for being with the patient and assisting them at the time you see them in clinic.**

Modified from Corwin Boake, Ph.D., TIRR, 1333 Moursund, Houston, TX 77030-3405, 713/799-6990
