# Supplementary material for: Development of the Fearless, Tearless Transition model of care for adolescents with an intellectual disability and/or autism spectrum disorder with mental health comorbidities
Source: Dev Med Child Neurol. 2020 Dec 17;63(5):560–5. doi: 10.1111/dmcn.14766 (PMC8247054; doi:10.1111/dmcn.14766)
Supplement: Supplementary file 8 — Appendix S5: 12‐year‐old checklist. [file DMCN-63-560-s004.docx]

| **UR:** | **Date:** | **Diagnosis:** |
| --- | --- | --- |

| Assessment Type | Description | Completed | Not Completed | N/A | Action Required / Notes |
| --- | --- | --- | --- | --- | --- |
| Does the patient have a GP | Refer to RCH Transition Service for providers  ***Ensure patient GP details are updated on EPIC*** |  |  |  |  |
| NDIS Application | If no application made application to be made with carer |  |  |  |  |
| Intellectual Assessment | Completion of intellectual/ADL assessment through school/private |  |  |  |  |
| HONOS-LD | **HONOS SCORE:** |  |  |  |  |
| Autism Parent Stress Index | **APSI SCORE:** |  |  |  |  |
| Supervision Rating Scale | **SRS SCORE:** |  |  |  |  |

|  | Mental Health Assessment for co-morbidities | | | | | | | | | | |
| --- | --- | --- | --- | --- | --- | --- | --- | --- | --- | --- | --- |
| Diagnosis | | **Completed** | **Not Completed** | **N/A** | **GP** | **Pediatrician** | **Psychiatrist** | **Psychologist** | **Speech Pathologist** | **OT** | **Other** |
| ASD | |  |  |  |  |  |  |  |  |  |  |
| ADHD | |  |  |  |  |  |  |  |  |  |  |
| Anxiety Disorder | |  |  |  |  |  |  |  |  |  |  |
| Depression | |  |  |  |  |  |  |  |  |  |  |
| Communication issues | |  |  |  |  |  |  |  |  |  |  |
| Mood Disorder | |  |  |  |  |  |  |  |  |  |  |
| OCD | |  |  |  |  |  |  |  |  |  |  |
| Psychosis | |  |  |  |  |  |  |  |  |  |  |
| Other (OT etc.) | |  |  |  |  |  |  |  |  |  |  |

| Carer Needs | | | |
| --- | --- | --- | --- |
| Focus | **Discussed** | **Not Discussed** | **Action Required / Notes** |
| Current Concerns |  |  |  |
| Current Supports / Needs |  |  |  |
